# Measles — California, January 1–April 18, 2014

**Published:** 2014-04-25

**Authors:** Jennifer Zipprich, Jill K. Hacker, Erin L. Murray, Dongxiang Xia, Kathy Harriman, Carol Glaser

**Affiliations:** 1 California Department of Public Health; 2 University of California, San Francisco School of Medicine

Measles is a highly contagious, acute viral illness that can lead to severe complications and death. Even patients who experience uncomplicated acute measles have a small risk for developing a devastating neurologic illness, subacute sclerosing panencephalitis, years after their infection. Measles was documented as eliminated (defined as interruption of continuous transmission lasting ≥12 months) in the United States in 2000 (1); however, importation of measles cases and limited local transmission continue to occur. During January 1–April 18, 2014, the California Department of Public Health received reports of 58 confirmed measles cases, the highest number reported for that period since 1995. Patients ranged in age from 5 months to 60 years. Three (5%) patients were aged <12 months, six (10%) were aged 1–4 years, 17 (29%) were aged 5–19 years, and 32 (55%) were aged ≥20 years. As of April 18, there had been 12 hospitalizations, and no deaths had been reported. During 2000–2013, the median annual number of measles cases reported in California was nine (range = four to 40).

Among the 58 cases, 54 (93%) were classified as importation-associated, including 13 importations, 13 cases epidemiologically linked to importations, 18 with virologic evidence suggesting recent importation, and 10 linked to cases with virologic evidence of recent importation.* The 13 importations were in U.S. residents who had returned from travel to the Philippines (eight), India (two), Singapore (one), Vietnam (one), and Western Europe (one). In contrast, in 2013, eight imported measles cases were reported in California, and none were from the Philippines. Forty-seven of the 58 cases were associated with 12 measles clusters (defined as two or more cases linked in time or place), which included nine outbreaks (defined as three or more cases linked in time or place). Transmission for 11 cases occurred in health-care settings; six of these 11 cases were in health-care personnel. Other transmission settings included households, a church day care center, an airplane, and a school.

Fifty-two of the 58 cases were confirmed by laboratory testing (44 by polymerase chain reaction and eight by immunoglobulin M), and six were confirmed by epidemiologic link to a laboratory-confirmed case. Genotypes identified were B3 (32 patients), the measles genotype currently circulating in the Philippines, and D8 (seven patients) (2,3). Five of the seven patients with D8 genotype reported international travel; the remaining two patients with D8 genotype were epidemiologically linked to the imported cases. Genotyping is pending for two of the four cases with unknown source of infection.

Most of the 58 patients were either unvaccinated (25 [43%]) or had no vaccination documentation available (18 [31%]). Of the 25 patients who were known to be unvaccinated, 19 (76%) had philosophical objections to vaccination, three (12%) were too young (aged ≤12 months) for routine vaccination, and three (12%) were unvaccinated for unknown reasons. Eleven (19%) patients had documentation of 2 or more valid doses of measles, mumps, and rubella (MMR) vaccine, including two children and nine adults. Three health-care personnel had documentation of serologic evidence of immunity before exposure, and one additional patient was found to have serologic evidence of immunity when tested as part of a contact investigation before symptom onset.

All persons who were exposed during travel were old enough to have received vaccination before travel (infants traveling to areas with endemic measles can be vaccinated at age 6–11 months). Five of the six unvaccinated travelers were unvaccinated because of philosophical objections; among these, one was not eligible to receive MMR vaccine at the time of travel because of pregnancy (4). Six adults had no vaccine documentation available, and one had received 2 valid doses.

In the United States, during January 1–April 18, 2014, a total of 129 cases of measles were reported, the highest number reported for this period since 1996. Among the cases were 34 importations, including 17 in travelers to and from the Philippines. The Philippines has been experiencing an explosive outbreak of measles, with approximately 20,000 confirmed or suspected cases reported during January 1–February 28 and 69 confirmed deaths (3). The increase in importations from this outbreak and subsequent transmission in certain settings in the United States highlights the importance of ensuring age-appropriate vaccination for persons traveling to areas where measles is endemic and maintaining high vaccination coverage at the national and local level.

All U.S. residents born after 1956 should ensure that they have received MMR vaccine or have serologic evidence of measles immunity. Vaccine recommendations for travel outside of North or South America by those born after 1956 who do not have serologic evidence of immunity include the following: 1 dose of MMR vaccine for infants aged 6–11 months and 2 doses of MMR separated by at least 28 days for children aged ≥12 months and adults (4,5). Prompt identification of patients with suspected measles and implementation of appropriate infection control can reduce transmission in health-care settings (6).

## Footnotes

* Additional information available at http://wwwn.cdc.gov/nndss/script/casedef.aspx?condyrid=908&datepub=1/1/2013%2012:00:00%20am.
